# Case of Crypsorchis

**Published:** 1885-07

**Authors:** Chas. S. Gwyn

**Affiliations:** Galveston, Texas


### CASE OF CRYPSORCHIS.

*By Chas. S. Gwyn, M. D., Galveston, Texas.*

For Daniel's Medical Journal.

Mr. M., aged 36 years, weight about 160 pounds.—To-day I was consulted for deformity of penis and apendages by Mr. M. Upon examination I found an infantile penis and scrotum without testicles, they being in the upper part of the inguinal canal. The history of the case, as far as I could learn, was that he was a "sevenmonths child," and was exceedingly small and delicate up to the age of eleven years, when his family physician applied a home-made truss with powerful springs, which gave him a great deal of pain, for a supposed double inguinal hernia, the irritation and probably inflammation caused occlusion of the lower portions of the inguinal canal, and the external abdominal ring preventing the descent of the testicles to the scrotum. The penis and scrotum were hardly larger than those of a four-year-old boy. There were a few, course, scattering hairs upon the pubes; no beard upon the face, hardly a down; voice strong and muscular, a deep bass, having regularly undergone the change at puberty (?); chest and limbs fully developed.

Complains that his passions and sexual desires give him both mental and physical pain, without the means to gratify them, yet he has erections of the rudementary organ, especially in his dreaming moments, so much so that he has to avoid female society as much as possible for the fear that "unclean thoughts" may trouble his repose.

He declines surgical interference.

